# The role of online hemodiafiltration with endogenous reinfusion in the treatment of systemic lupus erythematosus activity resistant to conventional therapy

**DOI:** 10.3389/fneph.2024.1269852

**Published:** 2024-03-22

**Authors:** Mohammed A. Elghiriani, Salah S. Naga, Ibtessam A. Hameed, Iman E. Elgohary, Amal R. Mansour

**Affiliations:** ^1^ Department of Internal Medicine, Faculty of Medicine, Alexandria University, Alexandria, Egypt; ^2^ Department of Clinical and Chemical Pathology, Faculty of Medicine, Alexandria University, Alexandria, Egypt

**Keywords:** systemic lupus erythematosus (SLE), C1q, online hemodiafiltration with endogenous reinfusion (HFR), systemic lupus disease activity index 2K (SLEDAI-2K), albumin/creatinine ratio (ACR)

## Abstract

**Introduction:**

Lupus is a diverse autoimmune disease with autoantibody formation. Lupus nephritis carries a grave prognosis. Complement involvement, namely, C1q deficiency, is linked to activity and renal involvement and could help in their assessment. LN therapies include plasma exchange, immune adsorption, and probably hemodiafiltration with online endogenous reinfusion (HFR), together with traditional immunosuppressive therapies.

**Aim:**

The aim of this study was to evaluate the role of HFR in improving signs and symptoms of systemic lupus erythematosus (SLE) activity and laboratory parameters in cases not responding to traditional immunosuppressive therapy.

**Settings and design:**

A controlled clinical study was conducted on 60 patients with lupus from Group A that was subdivided into two groups: cases 1 (47 patients), those who received traditional medical treatment, and cases 2 (13 patients), those who underwent HFR in addition to medical treatment. Group B consisted of two subgroups: control 1, composed of 20 healthy age- and sex-matched volunteers, and control 2, consisting of 10 cases with different glomerular diseases other than lupus.

**Methods and materials:**

Serum C1q was determined before and after the HFR as well as induction by medical treatment. Disease activity was assessed using SLEDAI-2K with a responder index of 50; quality of life was assessed using SLEQOL v2, and HFR was performed for the non-responder group.

**Results:**

C1q was lower in cases. It can efficiently differentiate between SLE patients and healthy controls with a sensitivity of 81.67% and a specificity of 90%. It can also efficiently differentiate between SLE patients and the control 2 group (non-lupus patients with renal glomerular disease) with a sensitivity of 83.33% and a specificity of 100%. C1q was more consumed in proliferative lupus, and correlated with anti-ds DNA, C3, and C4.

**Conclusions:**

C1q efficiently discriminates lupus patients and correlates with proliferative forms. HFR might ameliorate lupus activity and restore C1q.

## Introduction

Lupus can affect virtually every organ with a relapsing and remitting course. Women are affected more frequently than men (1). Presumed etiopathogenesis includes genetic, epigenetic, ethnic, immunoregulatory, hormonal, and environmental factors (2–5). Urine abnormalities with or without deranged laboratory parameters are common, and may eventually develop in up to 75% of patients with systemic lupus erythematosus (SLE) (3).

Complement involvement, namely, C1q deficiency, predisposes to SLE owing to a reduced ability to clear apoptotic cells. Genetic deficiencies of many classical pathways play a role in SLE (6, 7) Quantifying disease activity is very important to monitor and control. Many disease activity indices aid in monitoring and quantifying; among those indices is the Systemic Lupus Erythematosus Disease Activity Index (SLEDAI), which is based on the presence of 24 descriptors in nine organ systems; it has been used in both research and clinical settings. It is considered a predictive variable and outcome measure in prognostic studies of lupus (8, 9). SLEDAI-2K is a modification of SLEDAI to allow any ongoing disease activity in the descriptors: skin rash, alopecia, mucosal ulcers, and proteinuria. Therefore, SLEDAI-2K included any inflammatory rash, alopecia, or mucosal ulcers and new, recurrent, or persistent proteinuria >0.5 g/24 h (10).

Looking for new therapies is always a dynamic process to help serve that group of patients, and among those therapies are clearance-based approaches. Hemodiafiltration with endogenous reinfusion (HFR) is a dialytic method that combines the processes of diffusion, convection, and adsorption. It can adsorb proinflammatory cytokines and could improve LN prognosis through the counterbalance of the immune-modulatory response (11). There are a plethora of previous studies that confirmed its role in reducing inflammatory cytokines and even light chains. In this study, we tried to explore its role as one of the treatment armamentariums for SLE patients through its amelioration of ongoing inflammation and, subsequently, autoantibody generation given the relatively easy settings to perform it using a regular hemodialysis machine.

## Materials and methods

This study is a controlled simple manual randomized unblinded explanatory clinical study that was conducted in Alexandria University Hospitals after the approval of the ethical committee. Eligibility criteria included SLE patients in activity with lupus nephritis. The outcome was the improvement in signs and symptoms together with laboratory parameters of SLE patients in activity especially for patients who are resistant or intolerant to conventional therapy.

It included two groups (**Table 1**), Group A consisted of 60 SLE patients in activity, subdivided into 47 patients (cases 1) receiving the conventional immunosuppressive therapy (corticosteroids, mycophenolate mofetil, and cyclophosphamide) and 13 patients (cases 2) who underwent HFR in addition to medical treatment. Group B was subdivided into control 1, composed of 20 age- and sex-matched healthy volunteers who have no history of any disease, and control 2, composed of 10 cases with different glomerular diseases other than SLE; all patients were subjected to history taking and clinical examination plus routine laboratory investigations in addition to immune laboratory investigations and determination of C3, C4, anti-ds DNA, and C1q (12–17), which was done using enzyme-linked immunosorbent assay (ELISA) before and after treatment and HFR sessions. Disease activity and quality of life were assessed using both SLEDAI-2K (18) and SLEQOL (19), respectively.

Table 1Demographic data and baseline characteristics.

**Table:** 

	Cases (*n* = 60)		Control 1 (*n* = 20)		Control 2 (*n* = 10)		Test of sig.	*p*
	No.	%	No.	%	No.	%		
Sex								
Male	4	6.7	0	0.0	2	20.0	χ^2^ - 3.676	^MC^ *p* = 0.132
Female	56	93.3	20	100.0	8	80.0		
Age (years)								
Min–Max	15.0–66.0		19.0–41.0		18.0–56.0		*H* = 3.677	0.159
Mean ± SD	28.85 ± 10.68		30.0 ± 6.52		37.10 ± 14.42			
Median	27.0		29.50		42.0			
Marital state								
Single	28	46.7	14	70.0	5	50.0	*χ* ^2^ - 4.361	^MC^ *p* = 0.420
**Married**	**31**	**51.7**	**6**	**30.0**	**5**	**50.0**		
Divorced	1	1.7	0	0.0	0	0.0

**Table:** 

Obstetrics and gynecology	Cases		Control 1		Control 2		Test of sig.	p
	No.	%	No.	%	No.	%		
**Gravidity**	**(*n* = 31)**		**(*n* = 6)**		**(*n* = 5)**			
Min–Max	0.0–8.0		0.0–2.0		0.0–2.0		*H* = 8.853^*^	0.012^*^
Mean ± SD	2.58 ± 1.63		1.0 ± 0.63		1.40 ± 0.89			
Median	2.0		1.0		2.0			
**Sig. bet. grps**	*p*_1 = _0.006^*^, *p_2 = _* 0.129, *p*_3 = _0.411							
Parity								
Min–Max	0.0–6.0		0.0–1.0		0.0–2.0		*H* = 9.440^*^	0.009^*^
Mean ± SD	1.97 ± 1.17		0.83 ± 0.41		1.0 ± 0.71			
Median	2.0		1.0		1.0			
**Sig. bet. grps**	*p*_1 = _0.008^*^, *p*_2 = _0.051, *p*_3 = _0.695							
Abortion								
No	17	54.8	6	100.0	5	100.0	χ^2^ - 6.825^*^	^MC^ *p* = 0.029^*^
**Yes**	**14**	**45.2**	**0**	**0.0**	**0**	**0.0**		
1	10	71.4	0	0.0	0	0.0	–	–
2	2	14.3	0	0.0	0	0.0		
3	2	14.3	0	0.0	0	0.0		
Min–Max	0.0–3.0		–		–		–	–
Mean ± SD	0.65 ± 0.88		–		–			
Median	0.0		–		–			

Bold are the statistically significant values.

For Group A, 16 patients underwent renal biopsy (26.7%): 1 patient had inadequate (6.3%), 2 patients had class II (12.5%), 2 patients had class III (12.5%), 8 patients had class IV (50%), 2 patients had class IV+V (12.5%), and 1 patient had class VI (6.3%) biopsy findings.

The human C1q ELISA is an assay performed for the quantitative detection of human C1q. An anti-human C1q coating antibody is adsorbed onto microwells. Human C1q present in the sample or standard binds to antibodies adsorbed to the microwells; following incubation, unbound biological components are removed during a wash step and a biotin-conjugated anti-human C1q is captured by the first antibody. After incubation, the unbound biotin-conjugated anti-human C1q is removed during a wash step, and streptavidin-HRP is added and binds to the biotin-conjugated anti-human C1q antibody. Then, after yet another incubation, the unbound streptavidin-HRP is removed during a wash step, and substrate solution reactive with HRP is added to the wells; a colored product is formed in proportion to the amount of human C1q present in the sample or standard. The reaction was terminated by the addition of acid and absorbance was measured at 450 nm. A standard curve was prepared from seven human C1q standard dilutions and the human C1q sample concentration was determined.

Samples were collected in plain tubes, centrifugation was performed to obtain cell-free plasma, and the samples were aliquoted and stored frozen at −20°C until analyzed.

HFR (for non-responder cases) is a two-chamber single-use filter that employs separated convection, diffusion, and adsorption. In the convective phase (Synclear 02) of the first stage, pure ultrafiltrate (plasmatic water) passes through a sorbent cartridge containing hydrophobic styrene resin (Suprasorb^®^; Bellco Srl, Mirandola, Italy) consisting of numerous pores and channels that add to its extensive surface area, where adsorption takes place, and then finally diffusion ensues.

### Statistical analysis

Data were fed to the computer and analyzed using IBM SPSS software package version 20.0. (Armonk, NY: IBM Corp) (20, 21) Qualitative data were described using numbers and percent. The Kolmogorov–Smirnov test was used to verify the normality of distribution. Quantitative data were described using range (minimum and maximum), mean, standard deviation, and median. The significance of the obtained results was judged at the 5% level.

The following tests were used in the study:

1. Chi-square test

This was used to compare categorical variables between different groups.

2. Fisher’s exact or Monte Carlo correction

This was used as correction for chi-square when more than 20% of the cells have an expected count of less than 5.

3. Paired *t*-test

This was used to compare normally distributed quantitative variables between two periods.

4. Pearson coefficient

This was used to correlate between two normally distributed quantitative variables.

5. Mann–Whitney test

This was used to compare abnormally distributed quantitative variables between two studied groups.

6. Kruskal–Wallis test

This was used to compare abnormally distributed quantitative variables between more than two studied groups, and *post hoc* (Dunn’s multiple comparisons test) was used for pairwise comparisons.

7. Wilcoxon signed ranks test

This was used to compare abnormally distributed quantitative variables between two periods.

8. Spearman coefficient

This was used to correlate between two distributed abnormally quantitative variables.

9. Receiver operating characteristic curve (ROC)

It was generated by plotting sensitivity (TP) on the *Y* axis versus 1 − specificity (FP) on the *X* axis at different cutoff values. The area under the ROC curve denotes the diagnostic performance of the test. An area of more than 50% gives acceptable performance and an area of approximately 100% gives the best performance for the test. The ROC curve also allows comparison of performance between two tests.

10. Sensitivity

It shows the capacity of the test to correctly identify diseased individuals in a population (“true positives”). The greater the sensitivity, the smaller the number of unidentified cases (“false negatives”).

11. Specificity

It shows the capacity of the test to correctly exclude individuals who are free of the disease (“true negatives”). The greater the specificity, the fewer “false positives” will be included.

12. Positive predictive value (PPV)

It shows the probability of the disease being present, among those with positive diagnostic test results.

13. Negative predictive value (NPV)

It shows the probability that the disease was absent, among those whose diagnostic test results were negative.

### Sample size

A sample of 60 patients with SLE (22) is required to estimate an average change in signs and symptoms after HFR dialysis of 68% (23), with an effect size for new treatment regimen of 3.4 (23) with 95% confidence level and a study power of 80%. The sample size was calculated using PASS Software.

## Results

**Figure 1:**
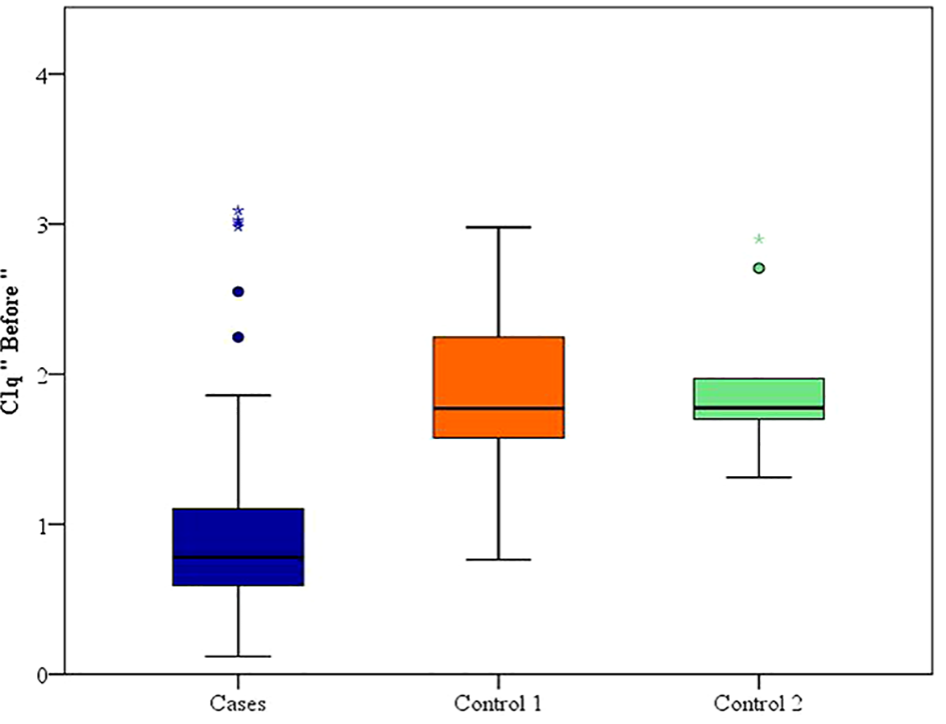
C1q can efficiently differentiate between SLE patients and both controls 1 and 2.

**Figure 2:**
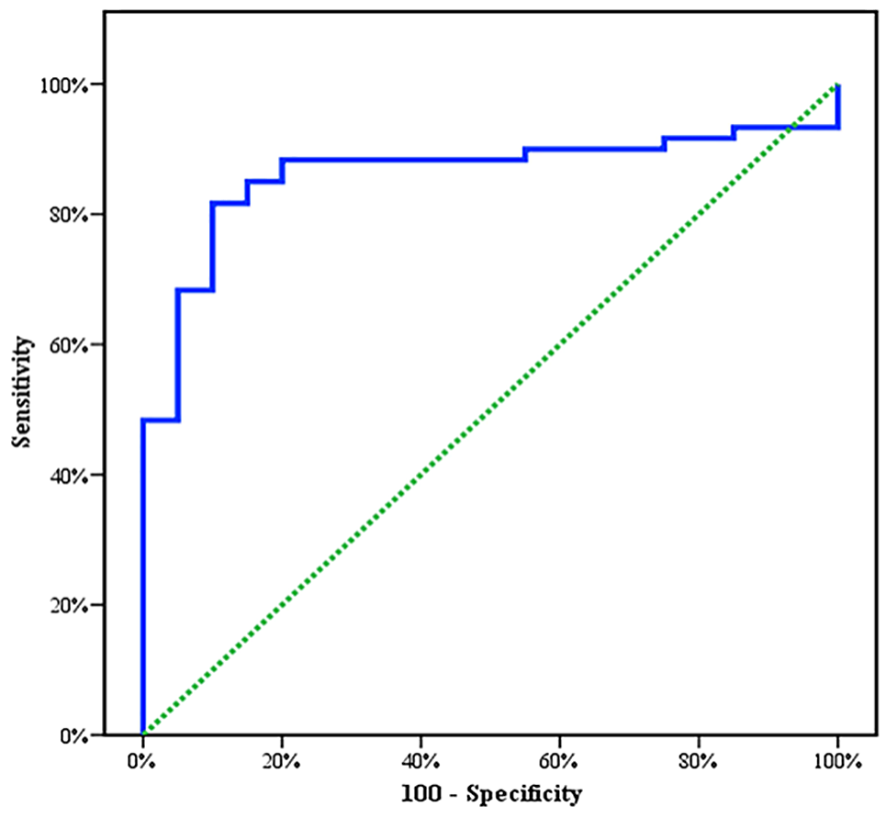
Agreement (sensitivity and specificity) for C1q to predict cases and control 1.

Between January 2018 and January 2020, among 100 patients in Alexandria University Hospitals enrolled in the study, 10 were excluded (due to refusal, sepsis, not meeting the criteria, and death). The recruited 90 patients were divided into Group A including 60 patients (subdivided into cases 1 and 2) and Group B including 30 patients (subdivided into controls 1 and 2) as depicted in the cohort diagram. (**Table 2**) C1q can efficiently differentiate between SLE patients and both controls 1 and 2 (**Figure 1**). The ROC curve for C1q in **Figure 2** shows that it can efficiently differentiate between SLE patients and healthy controls with a sensitivity of 81.67% and a specificity of 90% (**Table 3**); in addition, it shows that it can efficiently differentiate between SLE patients and control 2 (non-lupus patients with other glomerular diseases) with a sensitivity of 83.33% and a specificity of 100% (**Table 4**).

**Table 2:** Comparison between the three studied groups according to C1q “before”.

C1q “Before”	Cases (*n* = 60)	Control 1 (*n* = 20)	Control 2 (*n* = 10)	*H*	*p*
Min–Max	0.12–3.09	0.76–2.98	1.31–2.90	32.977^*^	<0.001^*^
Mean ± SD	0.98 ± 0.70	1.85 ± 0.55	1.93 ± 0.49		
Median	0.78	1.77	1.78		
**Sig. bet. grps**	*p*_1_ < 0.001^*^, *p*_2_ < 0.001^*^, *p*_3 = _0.765				

H: H for Kruskal–Wallis test, pairwise comparison between each of the two groups was done using post-hoc test (Dunn’s for multiple comparisons test).

p: p-value for comparing between the three studied groups.

p_1_: p-value for comparing between cases and control 1.

p_2_: p-value for comparing between cases and control 2.

p_3_: p-value for comparing between control 1 and control 2.

*: Statistically significant at p ≤ 0.05.

**Table 3:** Agreement (sensitivity, specificity) for C1q to predict cases and control 1.

	AUC	*p*	95% CI		Cutoff	Sensitivity	Specificity	PPV	NPV
			LL	UL					
**C1q**	0.863^*^	<0.001^*^	0.778	0.947	**≤1.168**	81.67	90.0	96.1	62.1

AUC, area under the curve p-value, probability value.

CI, confidence intervals *, Statistically significant at p ≤ 0.05.

**Table 4:** Agreement (sensitivity, specificity) for C1q to predict cases and control 2.

	AUC	*p*	95% CI		Cutoff	Sensitivity	Specificity	PPV	NPV
			LL	UL					
**C1q**	0.892^*^	<0.001^*^	0.816	0.967	**≤1.26**	83.33	100.0	100.0	50.0

AUC, area under the curve p-value, probability value.

CI, confidence intervals *, Statistically significant at p ≤ 0.05.

**Figure 3:**
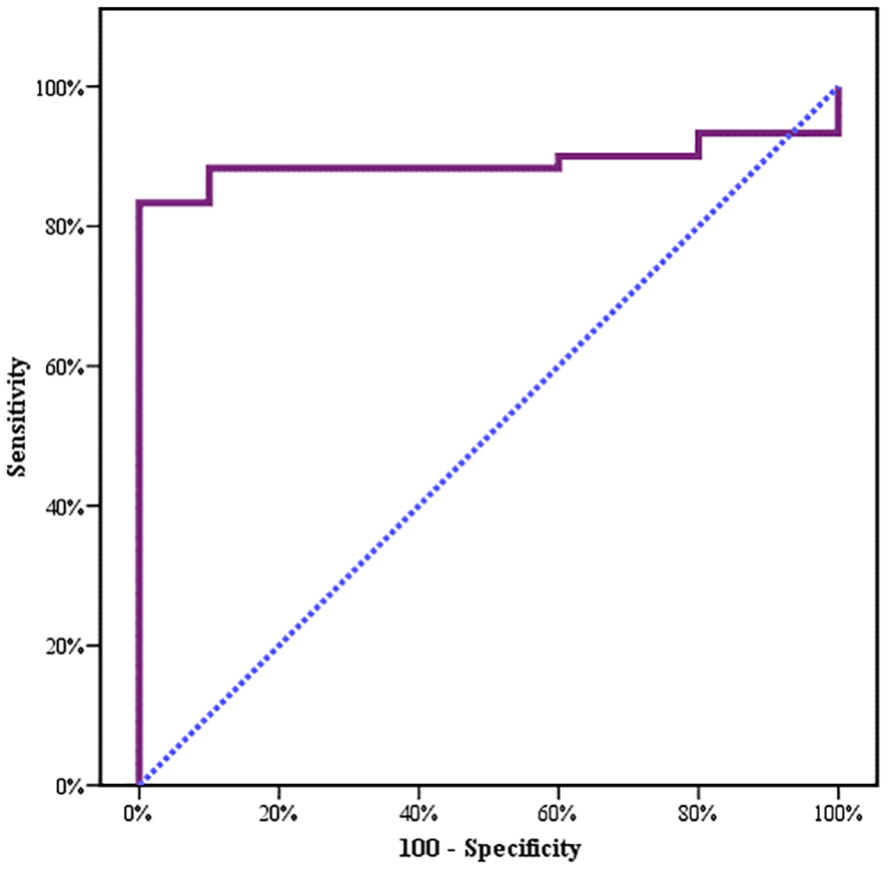
Second session of HFR showed a statistically significant difference in C1q levels before and after the session.

A difference with statistical significance was noted before and after medical treatment in the cases 1 group where C1q levels increased (**Table 5**). In the cases 2 subgroup, the second session of HFR showed a statistically significant difference in C1q levels before and after the session (**Table 6**) (**Figure 3**). Regarding SLEDAI-2K reduction, a statistically significant difference for both cases 1 and 2 before and after treatment was observed. A statistically significant difference was noted in the casse 1 subgroup regarding platelet count drop before and after medical treatment. Anti-ds DNA levels dropped significantly after medical treatment in cases 1. C3 levels increased significantly in cases 1 after medical treatment in comparison to before medical treatment.

**Table 5:** Comparison between before and after according to C1q medical treatment (cases 1).

C1q	Before (*n* = 47)	After (*n* = 47)	*Z*	*p*
Medical treatment				
Min–Max	0.12–3.09	0.08–2.66	1.979^*^	0.048^*^
Mean ± SD	0.92 ± 0.67	1.01 ± 0.59		
Median	0.75	0.89		

Z, Wilcoxon signed ranks test.

p, p-value for comparing between before and after.

*, Statistically significant at p ≤ 0.05.

**Table 6:** Comparison between before and after and HFR sessions according to C1q (cases 2).

C1q	Medical treatment		HFR sessions			
	Before	After	Before 1st session	After 1st session	Before 2nd session	After 2nd session
Medical treatment + HFR	(*n* = 13)	(*n* = 12)	(*n* = 12)	(*n* = 12)	(*n* = 6)	(*n* = 6)
Min–Max	0.17–3.01	0.10–3.01	0.10–3.01	0.11–2.94	0.46–1.18	0.61–2.26
Mean ± SD	1.18 ± 0.81	1.14 ± 0.82	1.09 ± 0.83	1.03 ± 0.74	0.77 ± 0.25	1.06 ± 0.61
Median	0.91	0.92	0.87	0.93	0.71	0.87
***Z*** (***p*)**	**0.447 (0.655)**		**0.235 (0.814)**		2.201^*^(0.028^*^)	

Z, Wilcoxon signed ranks test.

p, p-value for comparing between before and after.

*, Statistically significant at p ≤ 0.05. Bold are the statistically significant values.

A total of 16 patients had renal biopsy (26.7%): 1 had inadequate (6.3%), 2 had class II (12.5%), 2 patients had class III (12.5%), 8 patients had class IV (50%), 2 patients had class IV+V (12.5%), and 1 patient had class VI (6.3%) biopsy findings.

**Figure 4:**
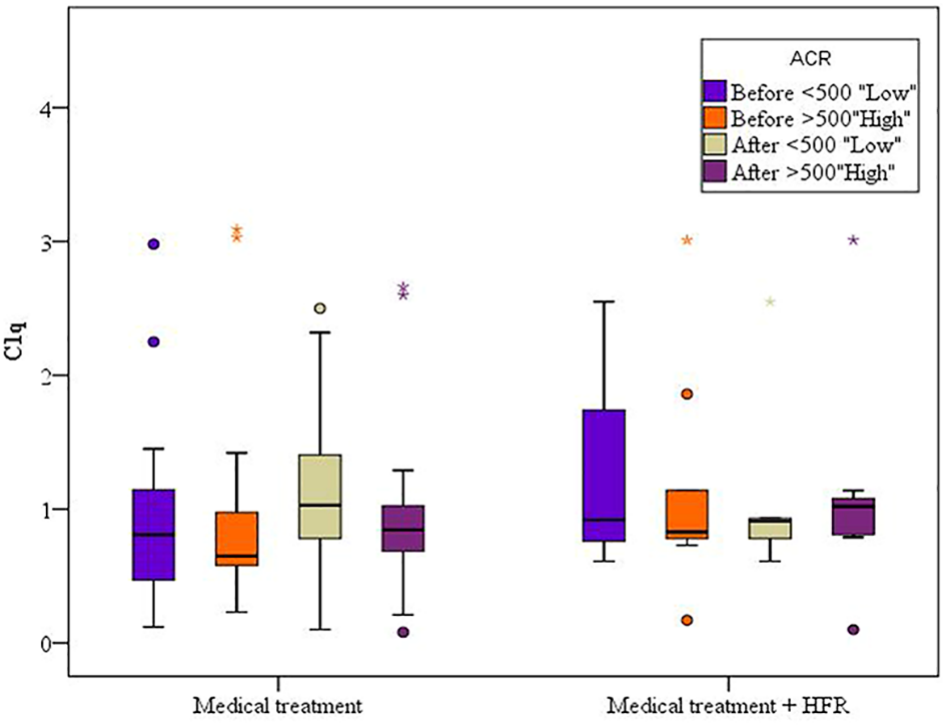
Cases 1 and cases 2 subgroups’ C1q values were lower when ACR exceeded 500 mg/g.

For the cases 1 subgroup, mean levels of C1q increased after treatment when compared to before treatment, and for the group of patients with an albumin/creatinine ratio (ACR) of less than 500 mg/g, mean values were 1.16 ± 0.60 (*p* = 0.085) and 0.96 ± 0.70 (*p* = 0.603), respectively, while for the subgroup of patients with an ACR of more than 500 mg/g, mean values were 0.92 ± 0.57 and 0.90 ± 0.67, respectively. For the cases 2 subgroup, mean levels of C1q decreased after treatment when compared to before treatment. In both cases 1 and cases 2 subgroups, C1q values were lower when ACR exceeded 500 mg/g, denoting possible correlation between C1q and lupus nephritis activity. However, the difference was statistically insignificant (**Figure 4**) (**Table 7**).

**Table 7:** Relation ACR and C1q in two groups of cases.

C1q	ACR (mg/g)			
	Before		After	
	<500 “Low”	>500”High”	<500 “Low”	>500”High”
Medical treatment “cases 1”	(*n* = 19)	(*n* = 28)	(*n* = 19)	(*n* = 28)
Min–Max	0.12–2.98	0.23–3.09	0.10–2.50	0.08–2.66
**Mean ± SD**	0.96 ± 0.70	0.90 ± 0.67	1.16 ± 0.60	0.92 ± 0.57
Median	0.81	0.65	1.03	0.84
***U* (*p*)**	**242.0 (0.603)**		**186.50 (0.085)**	
**Medical treatment + HFR “cases 2”**	**(*n* = 4)**	**(*n* = 9)**	**(*n* = 5)**	**(*n* = 7)**
Min–Max	0.61–2.55	0.17–3.01	0.61–2.55	0.10–3.01
Mean ± SD	1.25 ± 0.88	1.15 ± 0.83	1.15 ± 0.79	1.13 ± 0.90
Median	0.92	0.83	0.91	1.02
***U* (*p*)**	**17.0 (0.940)**		**14.0 (0.639)**	

U, Mann–Whitney test.

p, p-value for comparing between <500 “Low” and >500”High”. Bold are the statistically significant values.

Mean C1q values were low in classes III and IV with mean values of 0.98 ± 0.73 before and 1.02 ± 0.65 after treatment when compared to other classes except class VI, which was lower. This may be linked to C1q deficiency in proliferative lupus, despite it being statistically insignificant (**Table 8**).

**Table 8:** Relation biopsy and C1q in the case group.

C1q	Biopsy					*H*	*p*
	Inadequate (*n* = 1)	Class II (*n* = 2)	Class III + IV (*n* = 10)	Class IV + V (*n* = 2)	Class VI (*n* = 1)		
Before							
Min–Max		0.55–3.01	0.53–2.98	0.39–2.55			
Mean ± SD	0.57	1.78 ± 1.74	0.98 ± 0.73	1.47 ± 1.53	0.78	1.151	0.886
Median		1.78	0.72	1.47			
After							
Min–Max		0.80–3.01	0.08–2.66	0.81–2.55			
Mean ± SD	0.85	1.91 ± 1.56	1.02 ± 0.65	1.68 ± 1.23	0.78	1.915	0.751
Median		1.91	0.87	1.68			

H, H for Kruskal–Wallis test.

p, p-value for comparing between biopsy and C1q.

**Figure 5:**
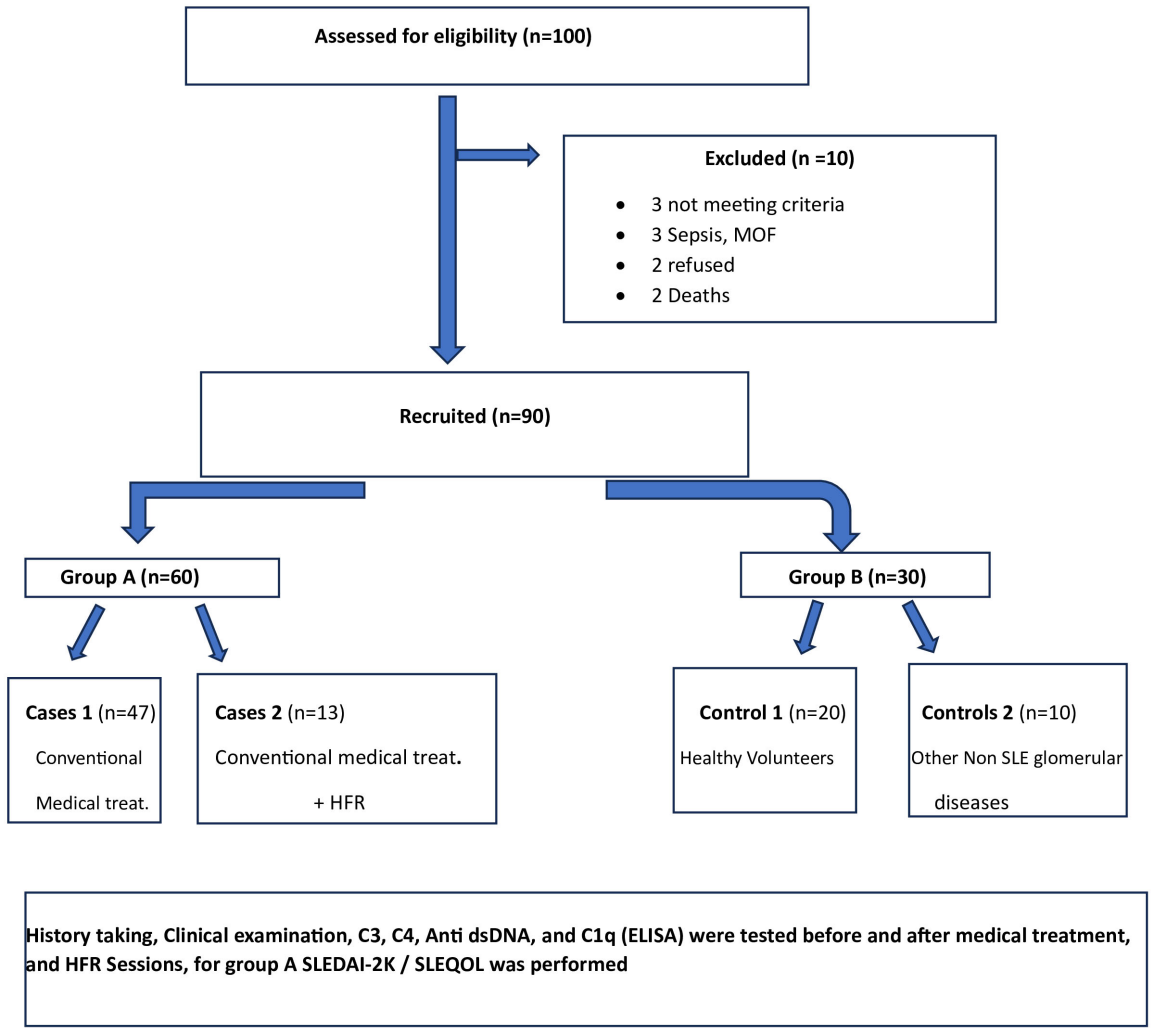
The study flow chart.

In cases, both C3 and C4 showed a significant positive correlation with C1q. Furthermore, a negative and significant correlation existed between C1q and anti-ds DNA. In the control 2 subgroup, there was a positive correlation between ACR and C1q. For the cases 1 subgroup before medical treatment, there was a statistically significant positive correlation between C1q and both C3 and C4, and a statistically significant negative correlation between C1q and anti-ds DNA. For the cases 2 subgroup after treatment, a significant positive correlation between C1q and SLEQOL was noted. For the cases 1 subgroup after treatment, there was a statistically significant negative correlation between C1q and anti-ds DNA. The study flow chart is depicted in **Figure 5**.

## Discussion

In the present study, we aimed to determine the role of HFR use in a subset of active lupus patients with and without lupus nephritis who are not responding to conventional therapy and/or intolerant to it. Complement system is of paramount role in SLE where C1q as an important complement component and activity marker especially for proliferative LN had highlighted that it could be a possible therapeutic target through its restoration and amelioration of complement activation.

We noticed a statistically significant difference between the case subgroups and both controls 1 and controls 2, regarding C1q; hence, it can significantly discriminate between SLE and both healthy populations as well as non-lupus causes of glomerular disease, and this supports the value of C1q and both the specificity and sensitivity of C1q to lupus patients (24–26).

In the Chinese study done by Pang et al. (27), they revealed that the specificity and prognostic yield for anti-C1q A08 antibodies were superior to either the intact or collagen regions of anti-C1q antibodies in disease relapse.

In our study, a significant difference before and after medical treatment in the case 1 subgroup was noted where C1q levels increased. In the second session of HFR, a statistically significant difference in C1q levels before and after the session was noted, and this may suggest the need for more than just one session of HFR. Thus, C1q is considered a marker of evaluation of activity and response to treatment. These findings may be supported and explained by the improvement in pro-inflammatory state in hemodialysis patients when they transitioned to HFR, as confirmed by reduced IL-6, IL-1β, and TNF-α and increased pre-albumin, in the study done by Borelli et al. (28).

Inflammation and cytokines represent a cornerstone in the etiopathogenesis of SLE; IL-6, retinol-binding protein 4 (RBP4), tumor necrosis factor α (TNF-α), IFN-α, and B-lymphocyte stimulator (BLyS) have all been detected in lupus as in the study done by Solano et al. (23).

Francesco et al. (27) used high-performance liquid chromatography coupled with a quadrupole time-of-flight mass spectrometer for the identification of proteins in the HFR session where ultrafiltrate and the species captured by the resin bed detected neutrophil gelatinase-associated lipocalin, zinc α-2 glycoprotein, and cystatin-C. Moreover, additional uremic toxins such as serotransferrin, β-2-glycoprotein 1, α-1-acid glycoprotein, prostaglandin-H2 D-isomerase, and transthyretin were identified. All of these markers are considered by many like Varghese et al. (29) as biomarkers for glomerular disease.

Moreover, Francesco et al. (23) reported fever and joint pain reduction, and improved skin manifestations and quality of life. After starting SUPRA (three times weekly for 4 h per session) for the patient case they studied, that patient no longer needed any additional plasma exchange treatment. Prednisone and immune suppressors were gradually reduced and eventually discontinued since the improvement of symptoms suggested a trend toward systemic remission. In this study, we had a patient who showed the same marvelous response, especially in the constitutional and musculoskeletal symptoms.

HFR was effective not only in SLE but even beyond, and it has been reported to reduce free light chain in myeloma kidney as revealed by Pasquali et al. (30) where they treated four patients affected by dialysis-dependent acute kidney injury due to biopsy-proven *de novo* free light chain myeloma cast nephropathy. Each patient received Bortezomib chemotherapy and extracorporeal treatment with the HFR. All patients had a considerable reduction in serum FLC, while serum albumin concentration remained unchanged. Renal function was restored in three out of four patients.

In this study, a statistically significant difference was noted in the cases 1 subgroup regarding platelet count drop before and after medical treatment. In the cases 2 subgroup, there was no statistically significant difference before and after medical treatment despite the fact that platelet count dropped.

Thus, whether HFR exerts a preserving effect on platelet count or function is not known. We had another case with persistent hemoptysis that was not responsive except to plasma exchange, and after the patient underwent HFR, he was free of hemoptysis for 2 weeks. One possible explanation for this is the amelioration of inflammation with subsequent reduction of pulmonary hemorrhage.

In both cases 1 and cases 2 subgroups, C1q values were lower when ACR exceeded 500 mg/g, denoting a possible correlation between C1q and lupus nephritis, despite being statistically insignificant.

Merete Bock et al. In Basel used anti-C1q antibodies as part of routine clinical care, and they aimed to assess the utility of anti-C1q antibodies as a marker of follow-up in lupus. They found that they correlate with activity scores as well as flare and remission and both urine protein creatinine ratio and anti-ds DNA levels, and negatively correlate with C3, C4, and CH50.

In agreement with our study, there was a negative insignificant correlation between ACR, SLEDAI-2K, SLEQOL creatinine, and C1q in the case subgroups. However, C3 and C4 showed a significant positive correlation with C1q. In addition, a negative and significant correlation existed between C1q and anti-ds DNA.

For cases 1 before treatment, there was a considerable negative correlation between C1q and anti-ds DNA, and a considerable positive correlation between C1q and both C3 and C4.

For cases 1 after treatment, there was a statistically significant negative correlation between C1q and both ACR and anti-ds DNA. For cases 2 after treatment, there was a statistically significant positive correlation between C1q and SLEQOL.

Mean C1q values were low in classes III and IV lupus nephritis before and after treatment when compared to other classes except class VI in which it was lower. This could be explained by a link between C1q deficiency and proliferative lupus. The study limitations included a relatively low sample size and the fact that follow-up for longer period would have given more insights into treatment effect. Study strengths are related to the easy, available setting for applying this triple-stage dialysis modality with convection, adsorption, and diffusion combined in one filter using a regular HD machine. It also offers a new treatment modality that needs further validation for SLE patients especially those with LN. To our knowledge, the study is internally and externally valid.

## Conclusions

C1q is a sensitive and specific diagnostic marker for lupus patients and may help as a marker of evaluation of activity and response to treatment; in addition, it is correlated with lupus nephritis. It is available, inexpensive, and non-invasive; thus, it may be of benefit for those who refuse or are contraindicated to do a biopsy, as well as to monitor for prediction of flare.

HFR could, through amelioration of inflammation, be a new therapeutic option for lupus patients, especially those with persistent disease activity despite traditional immunosuppressive therapy, and those who are intolerant to other therapies.

## Data availability statement

The raw data supporting the conclusions of this article will be made available by the authors, without undue reservation.

## Ethics statement

The studies involving humans were approved by Faculty of Medicine, Alexandria University. The studies were conducted in accordance with the local legislation and institutional requirements. The participants provided their written informed consent to participate in this study.

## Author contributions

ME: Writing – review & editing, Conceptualization, Investigation, Methodology, Project administration, Writing – original draft. SN: Writing – review & editing, Supervision. IH: Supervision, Writing – review & editing. IE: Supervision, Writing – review & editing. AM: Writing – review & editing, Supervision.
