# Supplementary figures and images for: The role of online hemodiafiltration with endogenous reinfusion in the treatment of systemic lupus erythematosus activity resistant to conventional therapy
Source: Front Nephrol. 2024 Mar 22;4:1269852. doi: 10.3389/fneph.2024.1269852 (PMC10995452; doi:10.3389/fneph.2024.1269852)

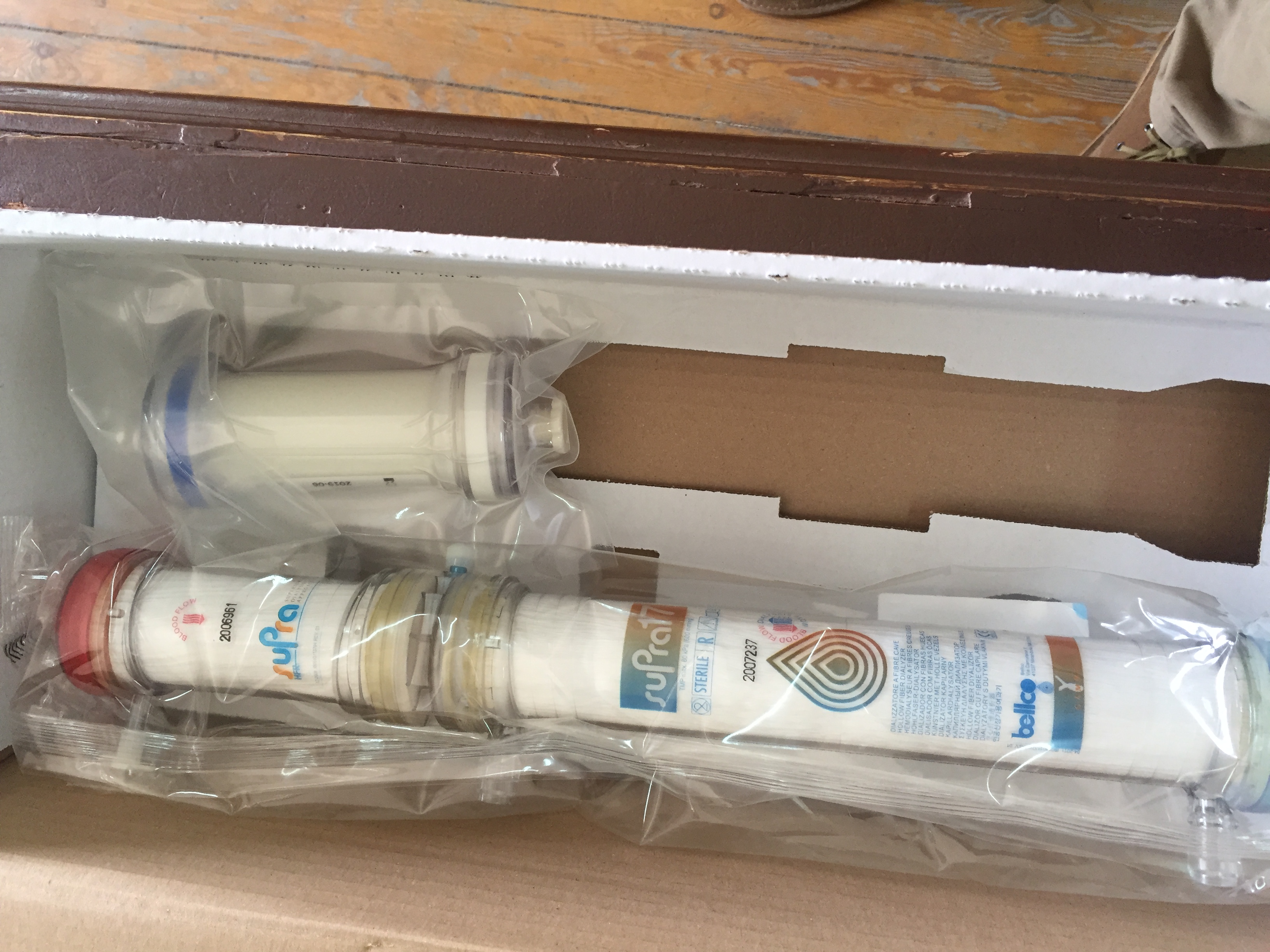

Supplement: Supplementary file 1 [file Image_1.jpeg]

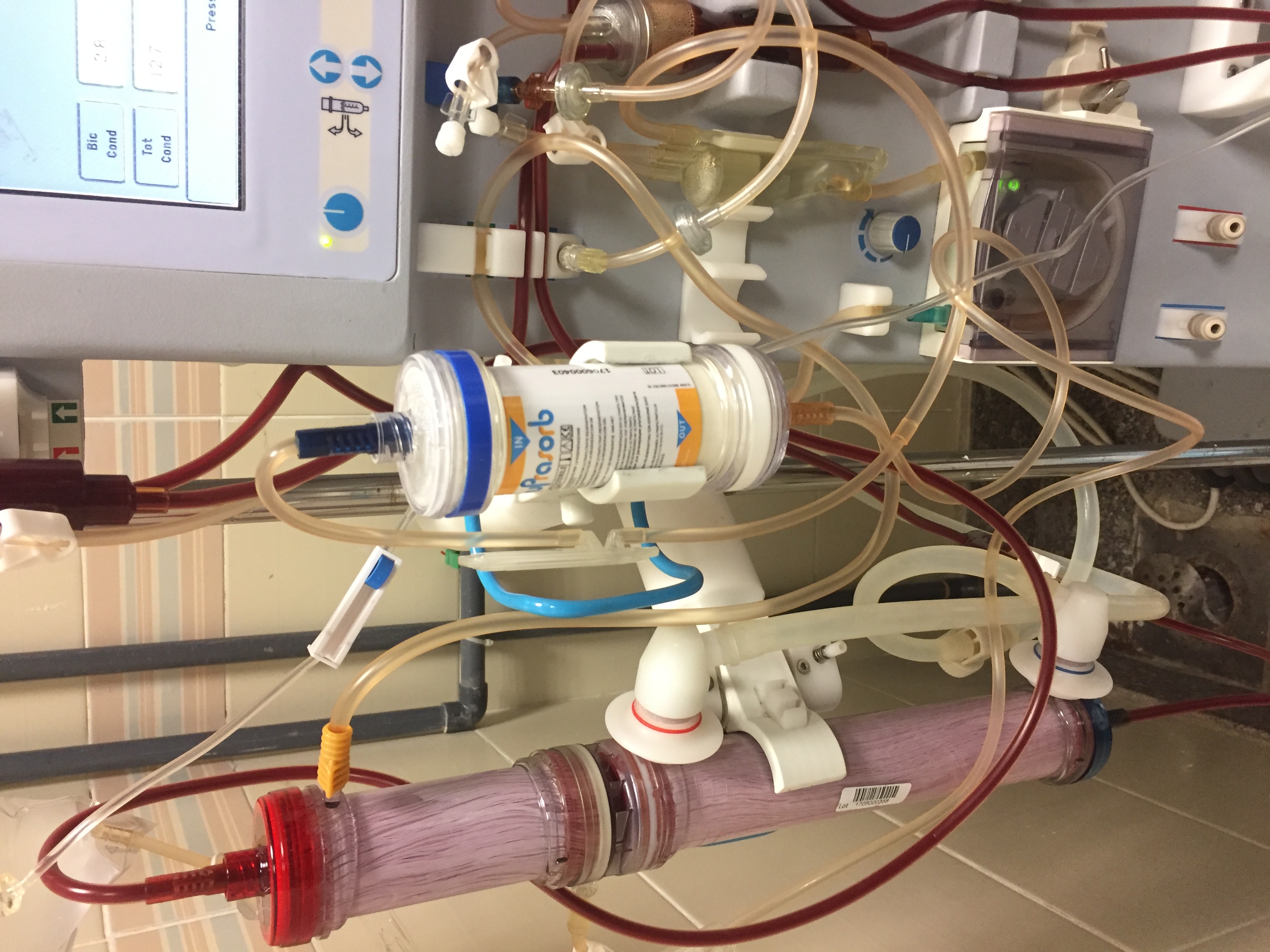

Supplement: Supplementary file 2 [file Image_2.jpeg]
